# Immunogenicity and Protective Efficacy of *Brugia malayi* Heavy Chain Myosin as Homologous DNA, Protein and Heterologous DNA/Protein Prime Boost Vaccine in Rodent Model

**DOI:** 10.1371/journal.pone.0142548

**Published:** 2015-11-11

**Authors:** Jyoti Gupta, Manisha Pathak, Sweta Misra, Shailja Misra-Bhattacharya

**Affiliations:** 1 Division of Parasitology, CSIR-Central Drug Research Institute, BS 10/1, Sector 10, Jankipuram Extension, Sitapur Road, Lucknow-226031, India; 2 Academy of scientific and Innovative Research, New Delhi, India; Instituto Butantan, BRAZIL

## Abstract

We earlier demonstrated the immunoprophylactic efficacy of recombinant heavy chain myosin (Bm-Myo) of *Brugia malayi (B*. *malayi)* in rodent models. In the current study, further attempts have been made to improve this efficacy by employing alternate approaches such as homologous DNA (pcD-Myo) and heterologous DNA/protein prime boost (pcD-Myo+Bm-Myo) in BALB/c mouse model. The gene *bm-myo* was cloned in a mammalian expression vector pcDNA 3.1(+) and protein expression was confirmed in mammalian Vero cell line. A significant degree of protection (79.2%±2.32) against L3 challenge in pcD-Myo+Bm-Myo immunized group was observed which was much higher than that exerted by Bm-Myo (66.6%±2.23) and pcD-Myo (41.6%±2.45). In the heterologous immunized group, the percentage of peritoneal leukocytes such as macrophages, neutrophils, B cells and T cells marginally increased and their population augmented further significantly following L3 challenge. pcD-Myo+Bm-Myo immunization elicited robust cellular and humoral immune responses as compared to pcD-Myo and Bm-Myo groups as evidenced by an increased accumulation of CD4+, CD8+ T cells and CD19+ B cells in the mouse spleen and activation of peritoneal macrophages. Though immunized animals produced antigen-specific IgG antibodies and isotypes, sera of mice receiving pcD-Myo+Bm-Myo or Bm-Myo developed much higher antibody levels than other groups and there was profound antibody-dependent cellular adhesion and cytotoxicity (ADCC) to *B*. *malayi* infective larvae (L3). pcD-Myo+Bm-Myo as well as Bm-Myo mice generated a mixed T helper cell phenotype as evidenced by the production of both pro-inflammatory (IL-2, IFN-γ) and anti-inflammatory (IL-4, IL-10) cytokines. Mice receiving pcD-Myo on contrary displayed a polarized pro-inflammatory immune response. The findings suggest that the priming of animals with DNA followed by protein booster generates heightened and mixed pro- and anti-inflammatory immune responses that are capable of providing high degree of protection against filarial larval invasion.

## Introduction

Lymphatic filariasis (LF), a neglected tropical disease caused by *Wuchereria bancrofti*, *Brugia malayi (B*. *malayi)* and *B*. *timori* affects ~1.4 billion people globally. Annual single dose mass drug administration (MDA) program faces threat of drug resistance [1] as already indicated in veterinary helminthes [2,3]. Discovery of new antifilarial molecules or a potent vaccine is required to overcome this constraint. Filariids possess a complex life-cycle where each life-stage interacts through distinct host immunological channel. Some individuals exhibit an aggressive immune response with development of pathological symptoms while majority with detectable blood microfilariae (mf) remain apparently asymptomatic with dramatically elevated IgG4, IL-10, TGF-β, specific T cell hypo-responsiveness with impaired IFN-γ and IL-2 production that may facilitate parasite survival [4–5]. Endemic normal subjects in spite of being parasite and symptom free, mount a polarized Th1 response suggesting a Th1 effector mechanism based protective phenomenon.

Irradiated L3, recombinant proteins [6–8] and subunit vaccines [9–11] have been employed in the past to discover safe and effective vaccine against human filariids. We earlier identified a few prophylactic candidate antigens [7,12] including *B*. *malayi* recombinant myosin (Bm-Myo) which showed reactivity with endemic normal bancroftian serum [13,14]. Myosin, a body wall muscle protein has been probed as a vaccine candidate in other nematode parasites as well [15]. Mysosin has been detected in the ES products after its release from the surface of parasite [16]. Immunization of rodents with a cocktail of Bm-Myo with another *B*. *malayi* recombinant protein (Bm-TPP) in our earlier study provided better protection over single protein via Th1/Th2 immunity [17].

DNA vaccines are third generation vaccines made up of a small, circular piece of bacterial DNA called plasmid which has been genetically engineered to produce one or two specific proteins (antigens) from a pathogen. DNA vaccine is the prime example of modern effective genetic immunization which entered human phase I and II trials in case of viral infections [18–23]. DNA vaccines are considered economic, they can be easily purified, protein remains in the native folded conformation and such vaccines have the advantage of inducing both humoral and cellular immune responses. Nevertheless, *in vivo* efficacy of genetic vaccines has not always been satisfactory. Several efforts have been employed to improve the efficacy of DNA vaccine such as codon/promoter optimization [24–26], use of adjuvants [27–28], formulations [29–30] and heterologous prime-boost regimes [31–32].

The heterologous DNA prime/ protein boost vaccination strategy is an effective method that may overcome the shortcomings of DNA vaccination by utilizing the benefits of DNA and protein vaccines to effectively induce both cell-mediated immunity and antibody responses against invading organisms. Filarial Chitinase, paramyosin, GST, tropomyosin, ALT-2 and SXP-1 have successfully been employed as experimental DNA vaccine [33–36] against LF.

Attempts have been made in the current investigation to generate protective immune response employing Bm-Myo as DNA, DNA prime/protein-boost in BALB/c mice and their immunoprophylactic efficacies have been compared with the purified recombinant myosin.

## Materials and Methods

### Animals

Male BALB/c mice (6–8 weeks old) were housed in our institute’s Laboratory Animal Facility, fed on standard pellet diet and water was provided *ad libitum* under pathogen free conditions.

### Ethics statement

All the animals and experimental procedures were duly approved by the Animal Ethics Committee of CDRI duly constituted under the provisions of CPCSEA (Committee for the Purpose of Control and Supervision on Experiments on Animals), Government of India. The study bears approval no. IAEC/2011/145 dated 03/07/2012.

### Plasmid construction

The The *bm-myo* gene (accession number AY705730) was amplified using 1 μM each of gene specific forward 5’GGATCCATGATGGATAATTCGGCACGAGAGTTC3’ and reverse 5’CTCGAGAAACTGTACGCGTGAAGAAGAACGGAG3’ primers, 200 μM each dNTP (Fermentas, USA), 0.5 unit Taq DNA polymerase (Invitrogen, USA), 1xPCR buffer and 1.5 μM MgCl_2_ under the conditions of initial denaturation at 95°C/10 min, 29 cycles at 95°C/45 s, 62.3°C/1.30 min, 72°C/2 min and one cycle at 72°C/10 min. The amplified product (1924 bp) was cloned in the mammalian expression vector pcDNA 3.1(+) (Invitrogen) at Bam HI/XhoI restriction sites and transformed in competent *Escherichia coli* (*E*. *coli*) DH5α cells. The transformants were screened for the presence of recombinant plasmids having *bm-myo* insert by colony PCR after restriction digestion. Fidelity of the cloned product was verified by sequencing. The recombinant pcDNA3.1-bm-myo (pcD-Myo) construct was maintained and propagated in DH5α cells. Endotoxin-free plasmid DNA was isolated by Endo-free plasmid isolation kit (Qiagen, Germany) and used for *in vitro* transfection and immunization studies.

### Transfection of plasmid constructs and Western blot

Vero cell line (Monkey kidney cells) was maintained in Minimal Essential Medium (MEM) (Invitrogen) fortified with antibiotic (penicillin 100 units/ml, streptomycin sulfate 100 μg/ml, neomycin mixture; Sigma, USA) and 10% Foetal bovine serum (FBS, Sigma). Vero cells were transfected with pcD-Myo construct using TurboFect transfection reagent (ThermoScientific, USA) following manufacturer’s protocol and protein expression was observed. Briefly, Vero cells were cultured in 24 well plates to produce 85–90% confluence before transfection. The diluted pcDNA3.1 vector and pcD-Myo construct (1 μg DNA each in 100 μl) were mixed with Turbofect (2 μl), incubated for 25 min at room temperature (RT) and the mixture was added drop wise to Vero cells under gentle rocking for transfection at RT for 45 min. Transfected cells were kept at 37°C in CO_2_ incubator for 4–6 h and after 24 h, the medium was replaced with the fresh medium containing 400 μg/ml gentamycin (Sigma). Stably transfected Vero cell lysate was electrophoresed on 10% SDS-PAGE and transferred to PVDF membrane which was incubated with primary antibody (1:1000 anti-Bm-Myo) and subsequently with secondary antibody (1:10,000 HRP-goat anti-rabbit IgG; Sigma). Primary antibody was earlier raised in a male rabbit (1kg) by subcutaneous (s.c.) administration of three doses of 100 μg each of Bm-Myo emulsified in Freund's Complete Adjuvant (FCA, Sigma) on day 0 followed by two booster doses of 100 μg each on days 15 and 30 in Freund's Incomplete Adjuvant (FIA).

### Expression and purification of recombinant protein

Bm-Myo cDNA clone was earlier picked up by immunoscreening of adult *female B*. *malayi* cDNA λuniZap expression library with human bancroftian serum pool and successfully cloned in pET28b expression vector, transformed in BL21 E. coli cells [14]. Transformants were grown in 10 ml Luria–Bertani (LB) medium containing 50 μg/ml of kanamycin overnight at 37°C. The overnight grown culture was inoculated in 500 ml of LB medium and further grown till A_600_ reached ~0.6. The culture was induced with 0.5 mM isopropyl-d-thiogalactopyranoside (IPTG, Sigma), re-incubated for another 3 h at 30°C to over-express the protein containing His tag at N-terminal. Induced cells were harvested, lysed in buffer A (50 mM NaH_2_PO_4_, 300 mM NaCl, pH 6.5) containing 10 mM imidazole and 1.0 mM phenylmethylene sulfonate fluoride(PMSF), sonicated and pelleted. The supernatant containing His-tagged Bm-Myo was passed through nickel nitrilotriacetic acid (Ni-NTA) affinity chromatography column. The column was washed with buffer-A containing 10 mM, 20 mM or 50 mM imidazole and the recombinant myosin was eluted with 250 mM imidazole. Elutes were analyzed on 10% SDS-PAGE and fractions containing purified protein were pooled, dialyzed overnight against 50 mM NaH_2_PO_4_ at 4°C. The protein content was determined by the Bradford method [13]. The endotoxin levels were determined by the LAL Chromogenic Endotoxin Quantitation Kit (Thermoscientific) following manufacturer’s protocol.

### Immunization in mice and L3 challenge

BALB/c mice were grouped as here under:

1. pcDNA (only vector)
2. Adjuvant control
3. pcD+Adjuvant control
4. pcD-Myo (recombinant plasmid construct)
5. Bm-Myo (recombinant myosin protein emulsified with adjuvant)
6. pcD-Myo+Bm-Myo (pcD-Myo plasmid construct and Bm-Myo as booster)

The immunization schedule is summarized in supplementary figures (S1 and S2 Figs). Sixty BALB/c mice (male, 18–22 g) were divided into 6 groups each containing 10 mice which were immunized on days 0, 15, 30 and 45. Control groups of mice (i, ii and iii) were injected with pcDNA (intradermally, i.d.), Adjuvant (FCA, FIA) (s.c.) or pcDNA+Adjuvant respectively. Group iv received 100 μg of pcD-Myo plasmid construct through i.d. route in 100 μl PBS (phosphate buffer saline pH 7.2). Group v received 25 μg of Bm-Myo+FCA (s.c.) on day 0 followed by three booster doses of Bm-Myo+FIA (s.c.) on days 15, 30 and 45. In the heterologous group vi, mice were primed with two i.d. injections of pcD-Myo construct on days 0 and 15 followed by two s.c. booster doses of Bm-Myo in adjuvant on days 30 and 45. On day 60, 5 mice from each group were randomly euthanized by intraval sodium (100 mg/kg) overdosing to measure the immune response including measurement of peritoneal exudate cell (PEC) population. Blood was collected for serum samples which were stored at—20°C for antibody measurement. The remaining 5 mice from each group were used for protection studies as described below as well as recruitment of various leukocytes in peritoneal cavity after L3 challenge. The experiment was repeated and the data is shown as mean±SE of two independent experiments using.

### Protection studies in BALB/c mice

A week following the last booster, five remaining mice from each group were intraperitoneally (i.p.) challenged with ~50 *B*. *malayi* L3 recovered from experimentally infected laboratory bred mosquitoes (*Aedes aegypti*). Mice were euthanized on day 10 post challenge and their peritoneal cavities were washed to recover the developing parasites [37].

### Bm-Myo specific serum IgG

IgG antibodies to Bm-Myo were determined in the mice sera by indirect ELISA [7]. Briefly, 96 well microtiter polystyrene plates (Nunc, Denmark) were coated with 1.0 μg/ml (100 μl/well) of Bm-Myo, plates were blocked with 3.0% skimmed milk in PBS, incubated at 37°C with serial two fold dilutions of primary antibody (serum from individual group of mice) and re-incubated with secondary antibody (1:10,000 HRP-goat anti-mouse IgG). The antigen-antibody reaction was detected after adding substrate OPD (o-phenyl diamine, Sigma) and the reaction was stopped with 2.5 M H_2_SO_4_. Readings were taken at 492 nm in a multiplate reader (Infinite Series M200, Tecan, Switzerland).

### Bm-Myo specific serum antibody isotypes

Antibody isotypes were measured by ELISA using antibody isotyping kit (Sigma) as mentioned earlier [7,13]. Wells were coated with 0.1 μg/ml of Bm-Myo, incubated with 1:1600 dilution of primary antibody, re-incubated with isotype-specific monoclonal antibodies (1:1000 dilutions of goat antimouse IgG1, IgG2a, IgG2b, IgG3, IgM and IgA) and subsequently with rabbit anti-goat IgG-HRP (1:5000).

### Antibody-dependant cellular adhesion and cytotoxicity (ADCC) *in vitro*

To determine the role of antibodies in larval killing, ADCC assay was performed as earlier described [13]. Briefly, ~10 L3 each were co-cultured at 37°C with 0.5×10^6^ PECs from naive BALB/c in triplicate in 96 well plate in presence of all groups of mice sera (optimized dilution 1:16) for 48 h and cell adherence and cytotoxicity to L3 observed. Cellular adherence was considered positive if >5 cells were attached to larval surface while limpid, damaged or immobile L3 were considered as dead.

Percentagecytotoxicity=Numberofdeadlarvae÷Totalnumberoflarvae×100.

### Isolation of PECs and Splenocytes

PECs from all immunized and control groups after immunizations as well as post L3 challenge were isolated in sterile ice cold PBS, using standard protocol. Briefly, small aliquot of PBS was injected into the mouse peritoneum with the help of a needle attached to a syringe and gently aspirated. The procedure was repeated until 8-10ml of peritoneal fluid was collected from each mouse. Thereafter, peritoneal fluid was centrifuged at 300xg for 10 min at 4°C and pelleted cells were washed twice with PBS and counted.

Spleens were aseptically removed, gently crushed, passed through a sterile nylon cell strainer of 70 mm pore size (BD Biosciences, USA) to obtain single cell suspension and erythrocytes were lysed with 0.84% chilled NH_4_Cl solution, cells were washed and suspended in complete RPMI (Sigma) [37].

### Reactive oxygen species (ROS)

Oxidative burst was measured in PECs by incubating 1×10^6^ cells with 1 μM DCF-DA (2-7-dichlorofluorescin diacetate, Sigma) for 15 min at 37°C in CO_2_ incubator. The cells were washed and ROS content was measured on FACS Calibur (BD Biosciences). Data was analyzed by Flow Jo software (Tree star Inc., USA) and mean ROS values were evaluated [38].

### Differential leukocyte population in peritoneal lavage after immunization and L3 challenge

2x10^6^ PECs from immunized as well as L3 challenged mice were divided in separate tubes and incubated with rat anti mouse monoclonal antibodies (BD biosciences) directed against CD3 (FITC; clone-45-2C11; T cell marker), CD19 (FITC; clone-1D3; B cell marker), Ly6C/G (PE-Cy7; clone-RB6-8C5; neutrophils marker), CD11c (PE-cy7 clone-HL-3 and singlec F (PE; clone-E50-2440; BD bioscience) as eosinophil markers and F4/80 (Pacific Blue; clone-BM8; invitrogen; macrophage marker). Cells were acquired on four or five-decade log-scale dot plots displaying forward scatter (FSC) area vs. side scatter area (SSC) after excluding the cell debris. Acquisition was carried out by FACS-Diva software on FACS Aria cell sorter (BD Biosciences) and analysis was performed using Flow Jo software (Tree star Inc., USA).

### Immunophenotyping of B cells and T cell subsets

Splenocyte suspensions (1x10^6^ cells) were probed with fluorochrome conjugated mouse monoclonal antibodies directed against CD4 (FITC; clone-H129.19), CD8 (phycoerythrin; clone 53–6.7) and CD19 (FITC; clone 1 D3) cell surface antigens following manufacturer’s protocol (BD Biosciences) as mentioned earlier [37]. Cells were incubated for 30 min at 4°C in dark, washed in PBS, suspended in FACS buffer and acquired on FACS after setting appropriate threshold voltages. A total of 20,000 events in the lymphocyte gate were acquired using BD cellquest software and data was analyzed by Flow Jo software (Tree star Inc.).

### Measurement of intracellular cytokines

4x10^6^ splenocytes were isolated and cultured with 5 μg of recombinant protein myosin for 12 h at 37°C in CO_2_ incubator followed by re-incubation with 10 μg/ml of Brefeldin A (BD Biosciences) in dark for 6 h at 37°C in CO_2_ incubator. Cells were washed and incubated with mouse seroblock FcR (BD Biosciences) for 10 min to block the non-specific binding of antibodies to CD16 and CD32 markers to curtail background signal. Cells were re-incubated in dark at 4°C overnight with FITC-CD4 antibody, washed with PBS, fixed and permeabilized for 15 min. Cell suspension was divided in separate tubes and incubated in presence of PE labelled anti-mouse monoclonal antibodies to IL-2, (clone-JES6-5H4), IL-4 (clone-BVD4-1D11), IFN-γ (clone-XMG1.2) and IL-10 (clone-JES5-16E3). Data was acquired on FACS Calibur and analyzed as above. PE labelled anti-mouse isotype control antibodies (IgG2b k for IL-2, IL-4, IL10 and IgG1 k for IFN-γ; BD Biosciences) were used as negative control.

### Statistical analysis

The data were analyzed by PRISM software (version 5.0) using one-way analysis of variance (ANOVA), Two-way ANOVA was employed wherever required and individual comparisons were made by Bonferroni method. Probability values (*P*) of <0.05, <0.01, <0.001 between immunized and control groups were considered as significant (*), highly significant (**) and very highly significant (***) respectively.

## Results

### *bm-myo* gene was cloned in mammalian expression vector pcDNA3.1(+) and transfected in Vero cell line with successful expression of protein

**Fig 1:**
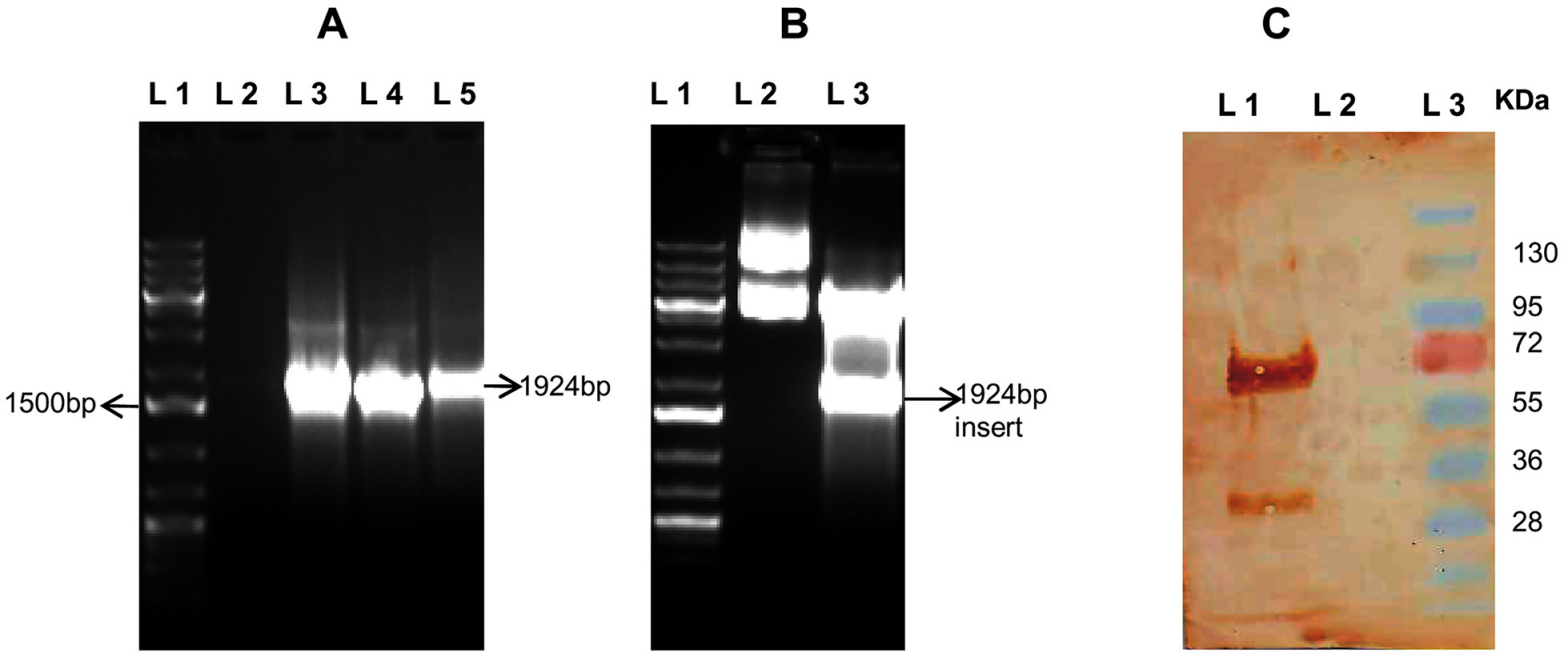
Cloning and expression of *bm-myo* in mammalian expression vector **(A)** PCR amplification of bm-myo. Lanes L1: 1Kb+ DNA ladder; L3-L5: amplified gene product **(B)** Confirmation of *bm-myo* gene insert in pcDNA3.1(+) construct. Cloning in pcDNA was verified by digesting the plasmid construct with endonucleases *BamHI* and *XhoI*.Lane 1: 1kb+ DNA ladder; Lane 2: undigested plasmid; Lane 3: *Bam HI* and *XhoI* digested pcD-Myo plasmid construct **(C)** Expression of Bm-Myo in transfected Vero cell line was carried out by anti Bm-Myo antibody. Lane 1: Western blot of pcD-Myo transfected construct; Lane 2: Western blot of only pcDNA transfected vector in Vero cell line, Lane 3: Standard protein molecular weight marker.

*bm-myo* gene was amplified from cDNA of adult *B*. *malayi* worms (Fig 1A), amplicon was cloned in the right frame of mammalian expression vector pcDNA3.1(+) and constructs were confirmed by restriction digestion (Fig 1B). Vero cells could be successfully transfected with pcD-Myo construct or vector pcDNA plasmid and protein expression was confirmed by Western blotting using rabbit anti-Bm-Myo antibody (Fig 1C). Sequencing confirmed no mutation demonstrating appropriately constructed recombinants expressed in mammalian cells.

### Bm-Myo was overexpressed as single band

~73 KDa recombinant Bm-Myo protein was successfully over expressed at optimal IPTG induction and purified as a single band from E. coli pET28b-BL-21 (DE3) through Ni-NTA affinity column after elution with 250 mM imidazole. The endotoxin level of the purified protein preparation was found to be <0.1 EU/μg.

### pcD-Myo+Bm-Myo conferred substantial protection against L3

**Fig 2:**
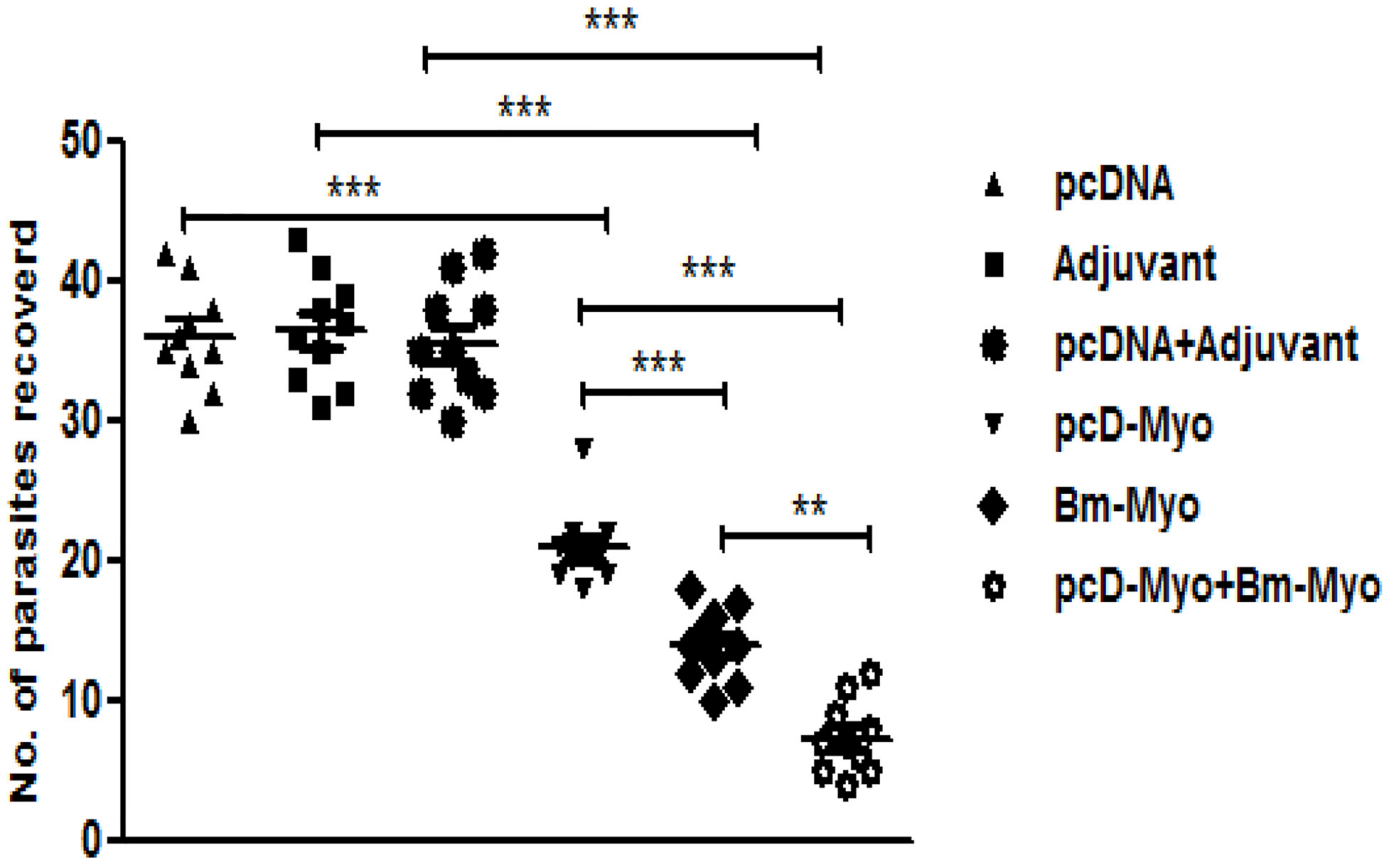
Mice were challenged with 50 L3 of *B*. *malayi* by intraperitoneal route and euthanized on day 10 post L3 challenge to recover developing parasites. The distribution graph was drawn by Prism 5.0 and horizontal bars represent arithmetic mean values derived from two separate experiments (n = 5) in each experiment. Significant difference in parasite recovery was obtained between pcD-Myo+Bm-Myo immunized group as compared to Bm-Myo (* *P*<0.01) and pcD-Myo (*** *P*<0.001). Similarly Bm-myo showed significant reduction in parasite recovery as compared to pcD-Myo (** *P*<0.01) group of mice.

Immunizations with pcD-Myo+Bm-Myo exhibited significant reduction (79.2%±2.32) in the recovery of developing larvae post L3 challenge as compared to Bm-Myo (*P*<0.01) and pcD-Myo (*P*<0.001). Bm-Myo and pcD-Myo led to 61.6%±2.23 and 41.6%±2.45 reductions in developing larvae recovered from mice on day 10 (Fig 2).

### Bm-Myo generated high levels of serum IgG

**Fig 3:**
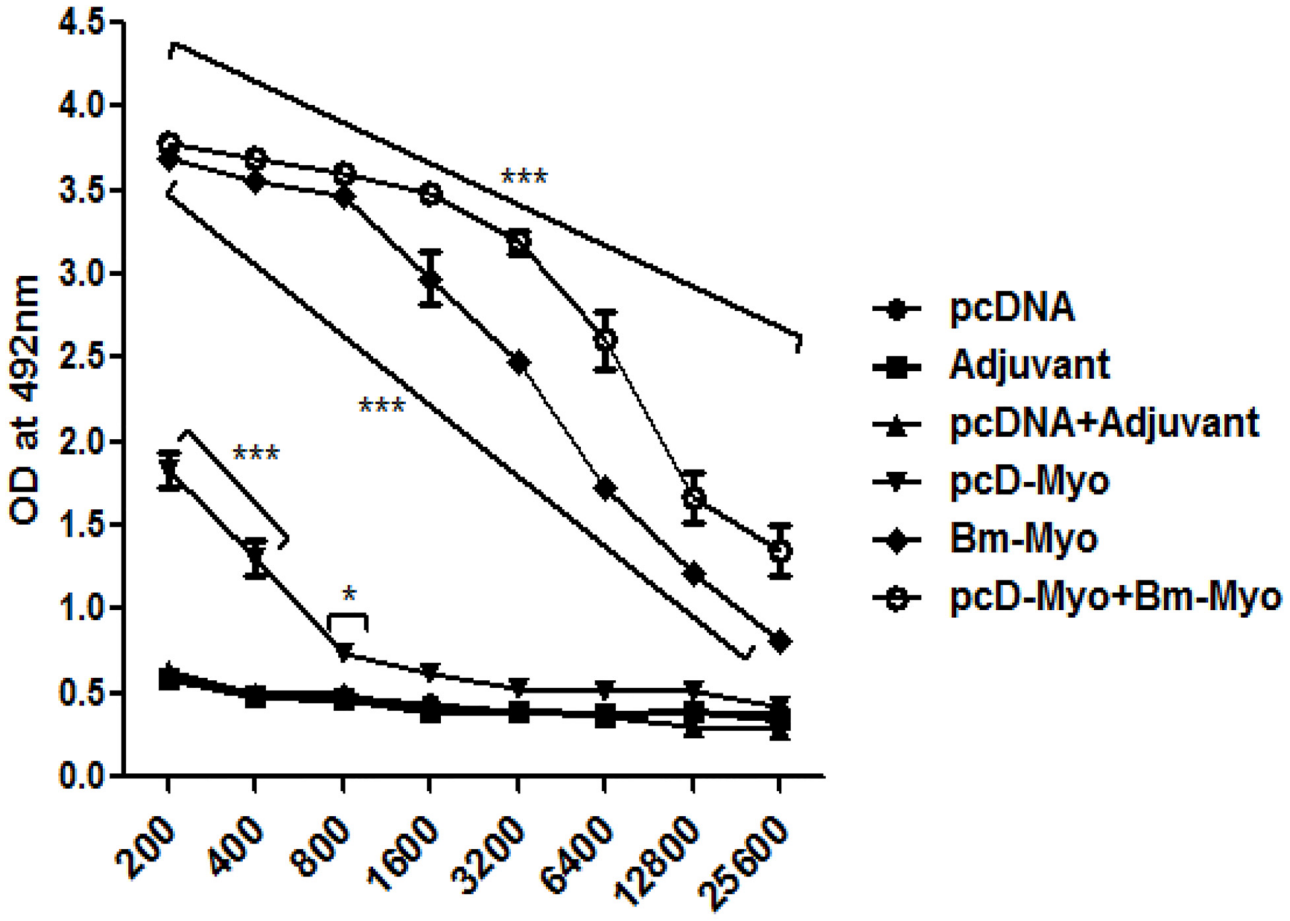
IgG titers were determined by indirect ELISA from serum obtained after last immunization booster. The IgG levels was significantly heightened (*** *P*<0.001) in pcD-Myo+Bm-Myo and Bm-Myo vaccinated mice at all dilutions over their respective controls (pcDNA+Adjuvant; Adjuvant) while pcD-Myo showed significantly increased IgG at lower dilutions (* *P*<0.05 to *** *P*<0.001) over pcDNA control group, shown in figure, howeve,r the difference was significant as compared to pcD-Myo+Bm-Myo and Bm-Myo (*** *P*<0.001 at dilution 1:1600 to 1:25600); pcD-Myo+Bm-Myo and pcD-Myo (*** *P*<0.001 at all dilution); Bm-Myo and pcD-Myo (*** *P*<0.001 at all dilution) (not shown in figure).

pcD-Myo+Bm-Myo administerd mice generated significantly higher titers of specific IgG antibodies at higher serum dilutions as compared to that of Bm-Myo group (*P*<0.001) (Fig 3) and at all dilutions with pcD-Myo immunized mice. Bm-Myo also triggered considerable IgG response (*P*<0.001) when comparison was made with pcD-Myo or other their respective control groups.

### pcD-Myo+Bm-Myo elicited a mixed Th1/Th2 response with marginal Th2 skewing

**Fig 4:**
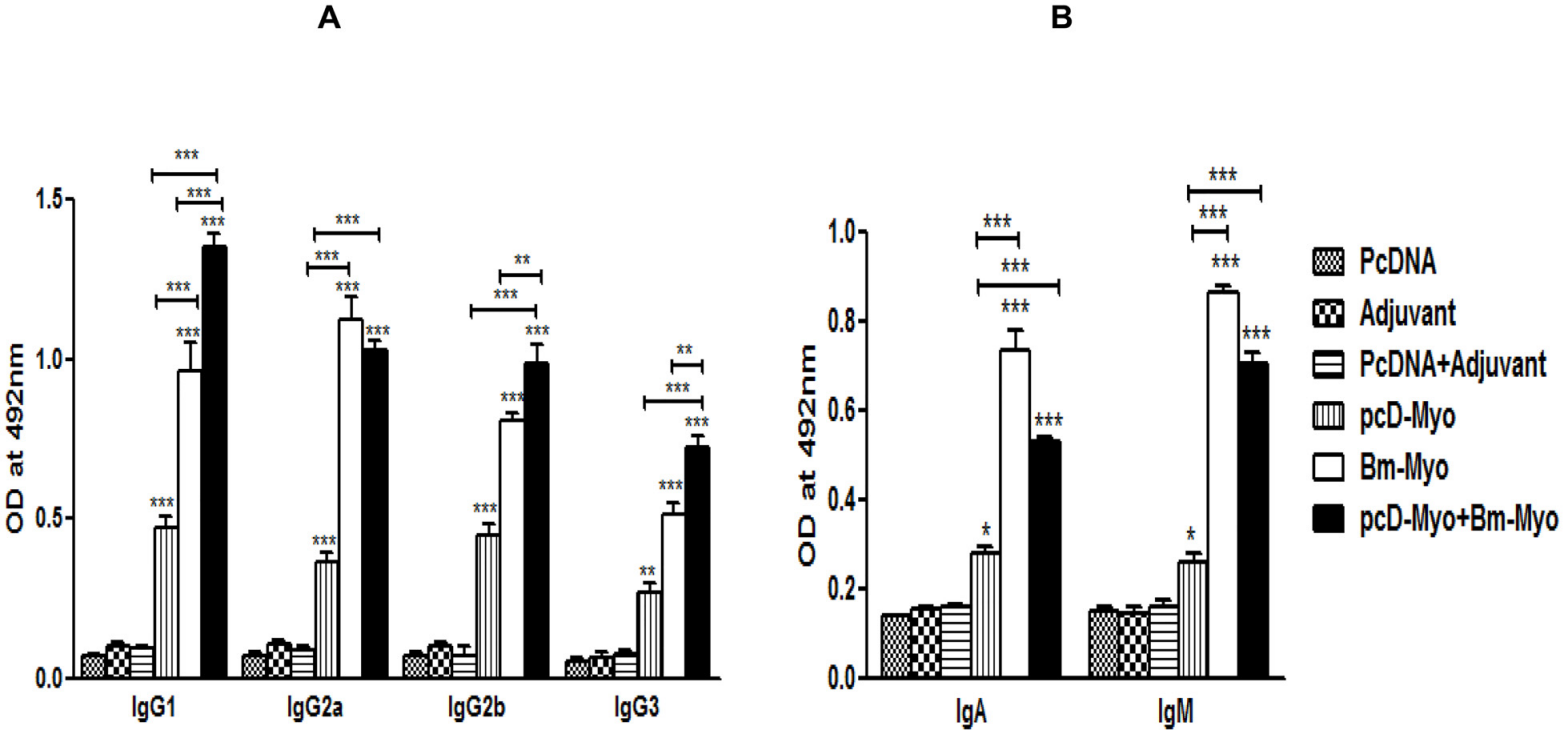
Bm-Myo specific antibody isotypes in the sera of mice. **(A)** Bm-Myo specific IgG1, IgG2a, IgG2b and IgG3 **(B)** Specific IgA and IgM antibodies in the serum samples of different groups of mice were measured by indirect ELISA at 1:1600 serum dilution. OD values (at 492 nm) are shown as mean±SE. Asterisks (*) on top of the bars represents statistical significance with respect to their controls (** *P*<0.01 and *** *P*<0.001 and above a line showed statistical significance with respect to each experimental immunized group (* *P*<0.05 and *** *P*<0.001).

All the three immunization schedules (pcD-Myo; Bm-Myo and pcD-Myo+Bm-Myo) led to generation of IgG1, IgG2a, IgG2b and IgG3 (*P*<0.001–0.001) (Fig 4A), however, pcD-Myo+Bm-Myo group of mice predominantly induced IgG1, IgG2a and IgG2b isotypes with IgG1:IgG2a ratio being >1 showing marginal bias towards Th2 immune response. Bm-Myo in contrast demonstrated a polarized Th1 response. Specific IgA and IgM were also elevated in pcD-Myo+Bm-Myo (*P*<0.001) and Bm-Myo (*P*<0.001) group while pcD-Myo administered animals produced high levels of IgA (*P*<0.01) instead of IgM (Fig 4B).

### Serum antibodies of pcD-Myo+Bm-Myo and Bm-Myo mice caused profound ADCC to L3

**Fig 5:**
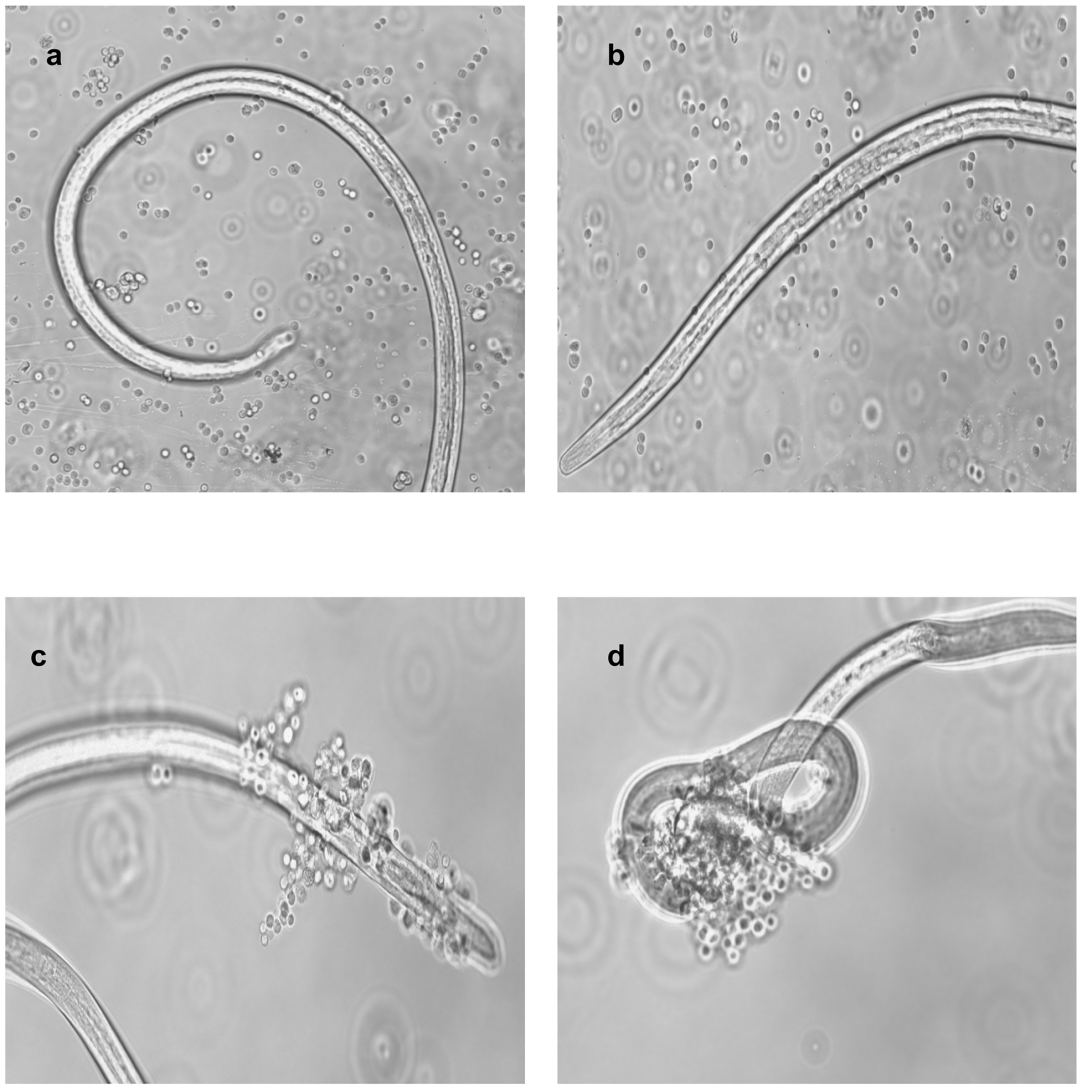
Antibody-dependent cell-mediated adhesion and cytotoxicity (ADCC). Presence of protective antibodies in the sera of (a) pcDNA (b) pcD-Myo (c) Bm-Myo (d) pcD-Myo+Bm-Myo immunized mice. Microscopic observation was recorded after 48 h by adherence of PECs on the surface of *B*. *malayi* L3 which ultimately may lead to their death. Photographs were captured on phase contrast fluorescent microscope (Nikon, Japan) at 40X magnification.

Serum of pcD-Myo+Bm-Myo and Bm-Myo mice promoted significant adherence of PECs to the surface of L3 causing 76.6%±0.50 and 64.3%±0.45 death of larvae within 48 h respectively in contrast to their respective controls (pcDNA+Adjuvant 20.0%±0.58; Adjuvant 23.3%±0.34). pcD-Myo or pcDNA did not show significant parasite killing (33.3±0.33% and16.3±0.30% respectively) (Table 1, Fig 5).

**Table 1:** Antibody-dependent cellular adherence and cytotoxicity (ADCC) to L3 using sera of BALB/c mice immunized with different formulations. ADCC assay was performed by incubating pooled mice sera (n = 5) samples with 0.5×10^5^ normal PECs and 10 *B*. *malayi* L3 at 37°C for 48 h. Sera from animals in each group were pooled and used in duplicate for the assay. Statistical significance of the differences between mean values of immunized and control groups are shown as * *P*<0.05 and *** *P*< 0.001. However, pcD-Myo+Bm-Myo showed significant difference over Bm-Myo (*** *P*<0.05) and pcD-Myo (*** *P*<0.001) while Bm-Myo increased percent cytotoxicity significantly over pcD-Myo (*** *P*<0.001), not shown in Table.

Experimental group	Percent cytotoxicity against L3
pcDNA	16.6±0.30
Adjuvant	23.3±0.34
pcDNA +Adjuvant	20.0±0.58
Myo-pcD	33.3±0.33*
Bm-Myo	64.3±0.45***
Myo-pcD+Bm-Myo	76.6±0.50***

* P<0.05, i.e., low significance;

***P<0.001,very high significance

### Increased recruitment of differential leukocyte population in the peritoneal cavity of mice

**Fig 6:**
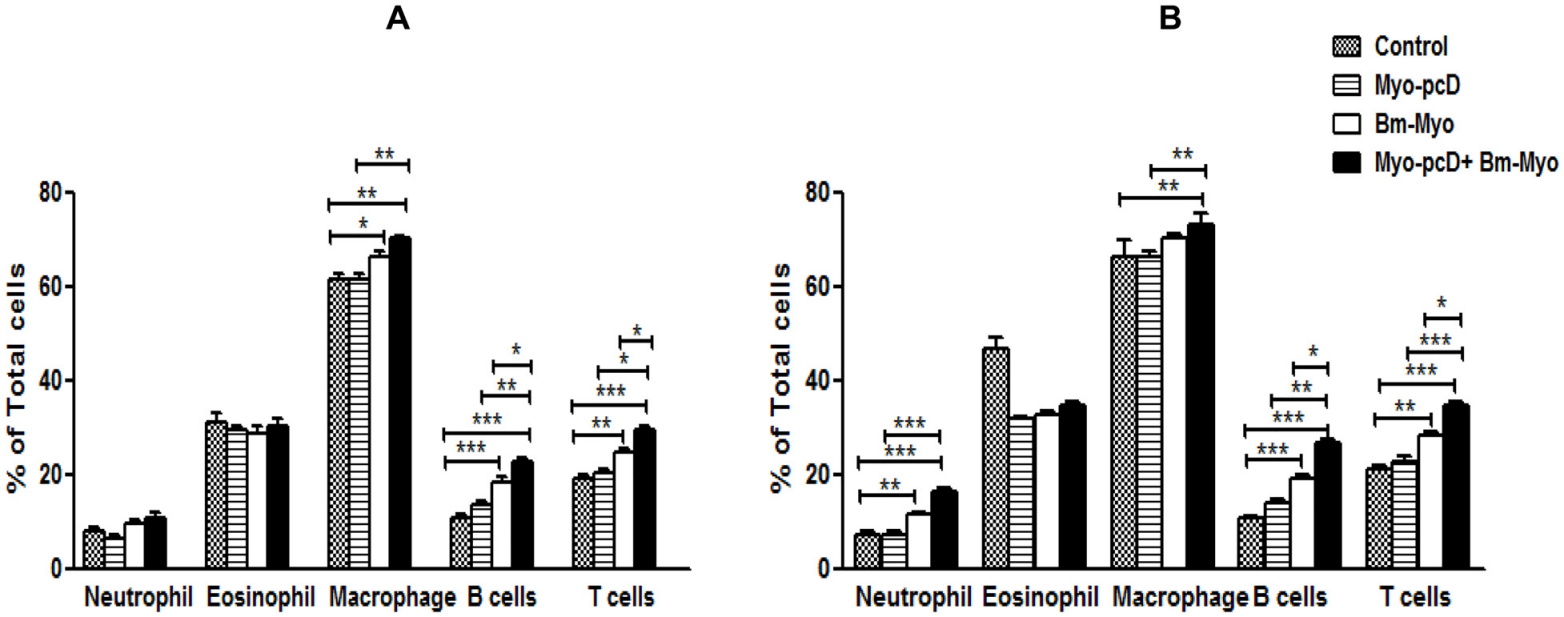
Differntial peritoneal leuckocyte population measured by flowcytometry: (A) After immunization (B) After L3 challenge. 2×10^6^ peritoneal cells from each group ofmice (n = 5 in each group) were isolated by lavage and were stained forLy6C/G, CD11c, singlecF, F4/80, CD3 as described in material method section and histogram representspercentages (mean±SE values) of different leukocytes as estimated by flow cytometry. Statistical significance between different groups is depicted as * *P*≤0.05; ** *P*≤0.01 and *** *P*≤0.001. However, pcDNA, Adjuvant and pcDNA+adjuvant control groups did not show any noticeable change in various cell populations so only one representative control group (pcDNA) shown in figure.

The peritoneal leukocyte population in all immunized as well as control mice were assessed after immunization and also post-L3 challenge by FACS as described in material method section. No significant differences were observed as regards to the percentages of neutrophils and eosinophils after immunization in different groups. An increased population of B and T lymphocytes were observed in pcD-Myo+Bm-Myo over DNA and protein groups (*P*<0.01 to *P*<0.05) while macrophages increased significantly (*P*<0.01) in this group when it was compared with Myo-pcD group. The increase in B cells, T cells and macrophage population was noticed in Bm-Myo group but it was statistically significant only in comparison to control group (*P*<0.01 to *P*<0.05) (Fig 6A). However, L3 infection further enhanced the cellular recruitment including neutrophils in the peritoneal cavity of mice and the differences were significant (P<0.05 to P<0.001) in between the animal groups shown in Fig 6B, while a comparison of peritoneal cell population post-immunization and post-L3 challenge in individual group revealed significant difference only in Myo-pcD+Bm-Myo group although other groups demonstrated marginal increase in different cell populations (S1 Table).

### Oxidative burst

**Fig 7:**
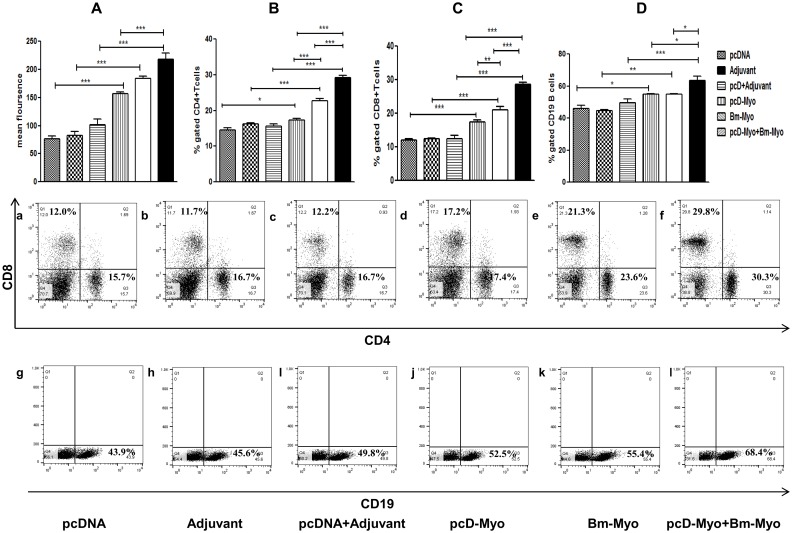
**(A)** Flowcytometric analysis of ROS generation by PECs. (1×10^6^/ml) cells isolated from all groups were loaded with probe DCFDA and ROS generation was evaluated. Values represent the mean fluorescence±SE. Flow cytometric analysis of T and B cells were carried out by staining of 1× 10^6^ cells with fluorescently labelled **(B)** CD4 **(C)** CD8 and **(D)** CD19 antibodies individually. Values represent the mean±SE from two separate experiments (n = 5 in each experiment). The differences in mean values were statistically analyzed and the comparison was done between all the immunized groups as well as their respective controls. The level of significance is depicted as * *P*<0.05; ** *P*<0.01 and *** *P*<0.001. Representative FACS dot plot of each group is shown in (a-l).

Real-time monitoring of oxidative burst in the peritoneal cells containing major population of macrophages revealed significantly higher generation of reactive oxygen species (ROS) in all the three groups of immunized mice (*P*<0.001). when compared with their respective controls (Fig 7A). However, pcD-Myo+Bm-Myo showed significant elevation in ROS generation as compared to pcD-myo but not in comparison to Bm-Myo.

### Heterologous prime boost profoundly induced T and B cell population in mouse spleen

Fluorescent labeled monoclonal antibodies to cell surface antigens could identify the target cell population by their phenotypic markers. However, all the three vaccine formulations enhanced the expansion of CD4+, CD8+ T cells and B cell population in mouse spleen as compared to their respective controls. PcD-Myo+Bm-Myo mice revealed major increase as compared to Bm-Myo or Myo-pcD (*P*<0.05 to <0.001) (Fig 7B, 7C and 7D). The representative dot plots are appended in the Fig 7(a–l).

### Production of both pro- and anti-inflammatory cytokines

**Fig 8:**
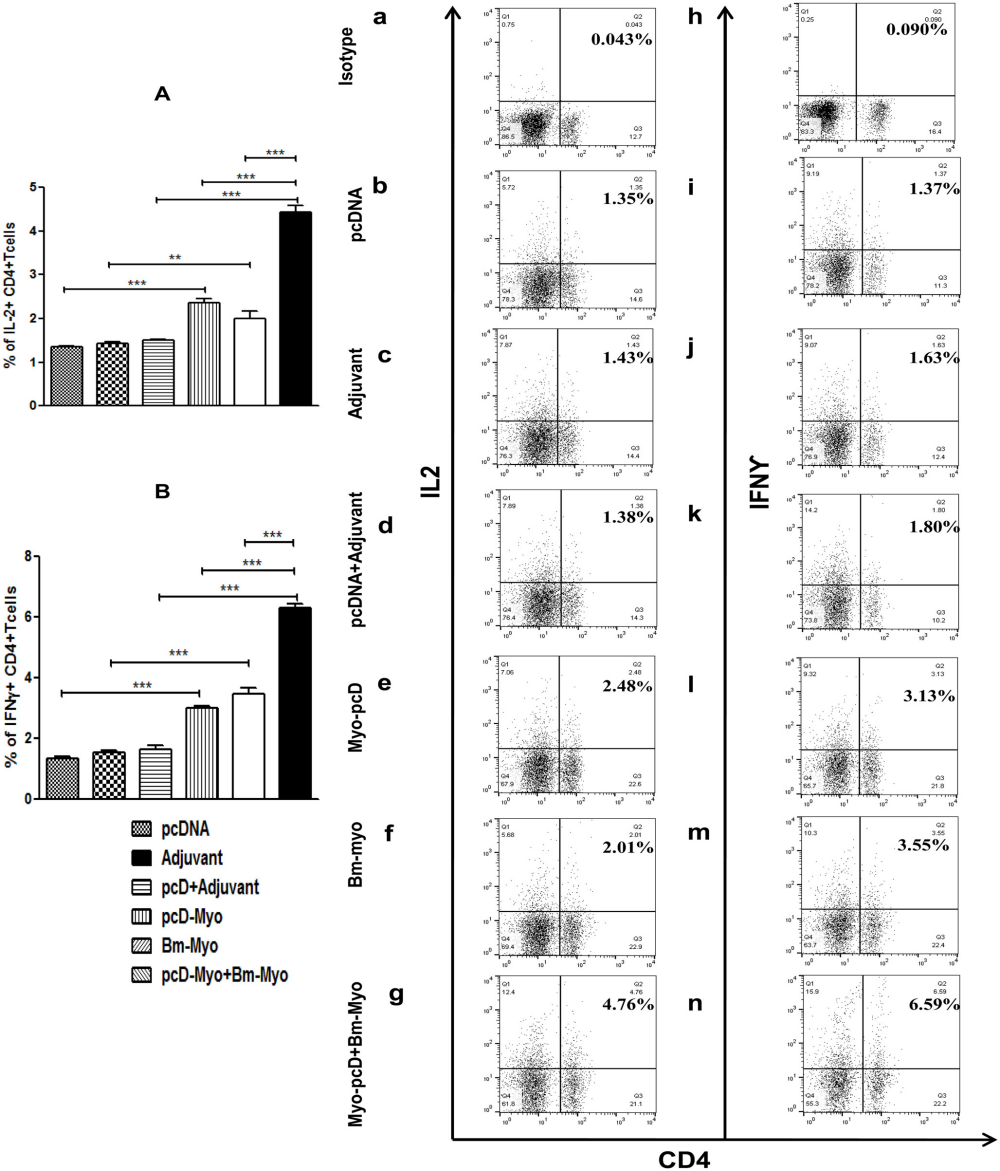
Measurement of pro-inflammatory (Th1) cytokines in mice spleen. Flow cytometric analysis of intracellular Th1 (IL-2, IFN- γ) cytokine production in CD4+ T cells. Splenocytes were stained and processed as described in the material and method section. Bar graphs were generated for exhibiting percentage of CD4+ T cells positive for (A) IL-2 (B) IFN-γ. Values represent the mean±SE from two separate experiments. Statistical significance of the differences between all immunized groups and their respective control groups are shown as * *P*<0.05; ** *P*<0.01 and *** *P*<0.001. Their representative FACS dot plots are also shown in figure (a-n). Numbers in the upper-right quadrant of each dot plot represents the mean percentage of CD4+ T cells positive for IL-2, IFN-γ in particular group. PE labelled anti-mouse isotype control antibodies (IgG2b k for IL-2 and IgG1 k for IFN-γ) were used a negative control to set quadrants.

**Fig 9:**
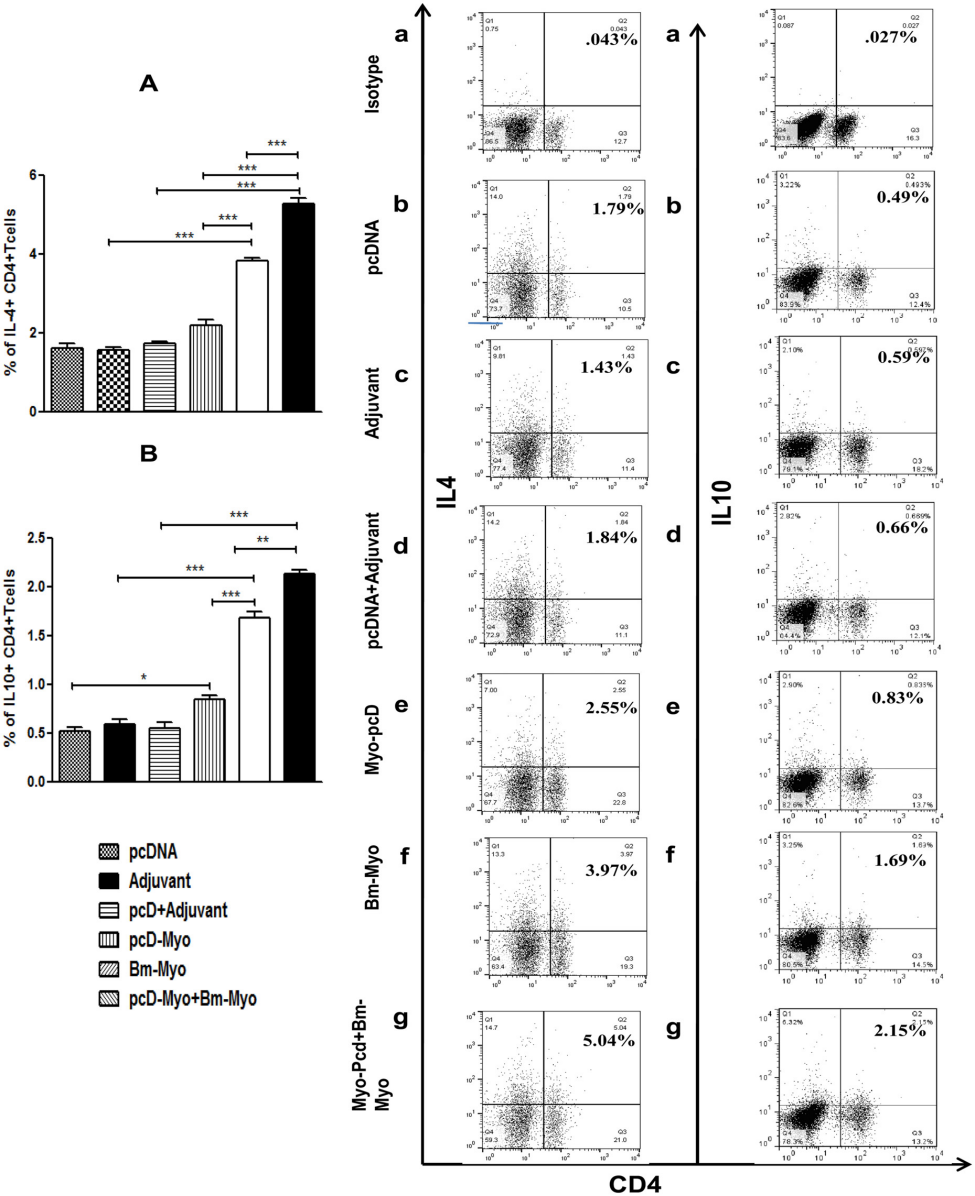
Measurement of anti-inflammatory (Th2) cytokines in mice spleen. Splenocytes were stained and processed as described in the material and method section and probed with fluorescently labelled monoclonal antibodies to IL-4 and IL-10 intracellular cytokines. Bar graphs were generated for exhibiting percentage of CD4+ T cells positive for **(A)** IL-4 and **(B)** IL-10. Values represent the mean±SE from two separate experiments. Statistical significance of the differences between mean values of all immunized as well as their respective control groups is shown as * *P*<0.05; ** *P*<0.01 and *** *P*<0.001 Representative FACS dot plots are shown in (a-n). PE labelled anti-mouse isotype control antibody (IgG1 k) was used a negative control to set quadrants.

Administration of pcD-Myo+Bm-Myo and Bm-Myo produced both pro-inflammatory (Th1) (IL-2, IFN-γ) (Fig 8A and 8B) and anti-inflammatory (Th2) (IL-4 and IL-10) cytokines (*P*<0.001) (Fig 9A and 9B) coinciding with an up-regulated CD4+ T lymphocyte population. PcD-Myo promoted enhanced production of IL-2, IFN- γ and IL-10 (*P*<0.001 to *P*<0.0), however, it had no significant effect on IL-4. The representative FACS dot plots are shown in Figs 8(a–n) and 9(a–n) and data suggest that Bm-Myo provides significant Th1 and Th2 immune response in mice, however, CD4+ population producing these cytokines was much higher in the pcD-Myo+Bm-Myo group compared to other two regimens (*P*<0.001) suggesting their possible role in anti-larval protection.

## Discussion

We earlier picked up Bm-Myo cDNA clone by immunoscreening of adult female *B*. *malayi* cDNA λuniZap expression library and demonstrated its potential as an immunoprophylactic candidate antigen in single [13] as well as cocktail immunization with two other *B*. *malayi* recombinant proteins; independent phosphoglycerate mutase (Bm-iPGM) and trehalose-6-phosphate phosphatase (Bm-TPP) in rodent model [17]. An attempt has been made in the current investigation to elaborate on the potency of identified vaccine candidate (Bm-Myo) employing alternative immunization approaches such as homologous DNA and heterologous DNA prime/protein boost vaccination. DNA vaccination is one of the attractive approaches of vaccination that may able to facilitate expression of antigens that would be closer to native epitopes unlike recombinant proteins. The DNA vaccines remain poorly immunogenic compared to protein vaccines [39], however, new findings suggest that the prime-boost can be more immunogenic than the homologous vaccination and may elicit unique immune responses allowing for improved immunogenicity and protection against viral, bacterial, and parasitic infections [40–44].

In the current investigation, the pcD-Myo construct was successfully cloned in pcDNA 3.1 mammalian vector and expressed in Vero cell line and the immunogenicity and protective efficacy of pcD-Myo and pcD-Myo+Bm-Myo was evaluated in BALB/c mice and compared with homologous recombinant protein Bm-Myo. The protection studies against L3 challenge showed that pcD-Myo+Bm-Myo offered best protection (~79%) followed by Bm-Myo (~62%) unlike pcD-Myo (~42%) construct alone. Thus it becomes apparent that priming of mice with DNA construct needed further protein boosters to generate higher extent of protective efficacy. In heterologous prime boosts, the encoded antigen is delivered in a different form, which may facilitate focusing of the response on desired foreign antigen by minimizing distraction and antigenicity of the delivering vector [45]. Our results of humoral response showed that IgG antibody and IgG isotypes were remarkably much higher in mice immunized with pcD-Myo+Bm-Myo demonstrating that DNA priming followed by protein boosting achieved greater humoral immune responses than does either DNA or protein given separately [46–48]. There are sufficient evidences to show that IgG1, IgG2 and IgG3 antibodies provide adequate protection against LF in humans and mice [49–51]. Since all the three vaccinations up-regulated the production of both IgG1 and IgG2a isotypes indicating the production of mixed T helper cell response, However, the ratio of IgG1/IgG2a in each immunization group varied which was marginally biased towards Th1 type in Bm-Myo group as also mentioned earlier [13], while leaned towards Th2 in pcD-Myo+Bm-Myo administered group of mice. There are reports demonstrating production of humoral response predominantly of Th2-type after intradermal injection or topical application of immunogens [52]. Nevertheless, antigen-specific cytokine profile as observed by flow cytometry did not truly reflect this pattern and both Th1 and Th2 cytokines appeared equally elevated as also reported in other previous studies [53]. IgG1, IgG2a and IgM antibodies are suggested to be instrumental in killing of *B*. *malayi* L3 via ADCC mechanism, corresponding to this, our study demonstrated that pcD-Myo+Bm-Myo promoted adherence of PECs to the surface of L3 resulting in larval immobilization and death. Not much information is although available on the role of IgA in protection against helminths though high IgA levels have been significantly correlated with the absence of filarial infection [54].

The immunized animals were challenged with *B*. *malayi* L3 in the peritoneal cavity and therefore differential leukocyte population in the peritoneal cavity of mice were analyzed which affirmed the crucial role of these cells in parasite clearance. The data revealed that Myo+Bm-Myo and Bm-Myo immunization up-regulated the percentage of macrophages, B and T lymphocytes which enhanced further significantly following L3 challenge. Neutrophils showed an increase especially after larval challenge suggesting their effective involvement in killing of larval parasites and indicated a complex interaction of these cells at the site of infection. Earlier reports also indicate that various cell population of peritoneum play a vital role in parasite killing and rapid accumulation of B cells and T cells is crucial to increase the number of macrophages and other cells that might have significant functional implications in the defense against *Brugia* infections [55–56]. There was only a marginal increase in the percentage of eosinophils in immunized mice post-L3 challenge in contrast to L3 infected control animals which demonstrated considerably increased infiltration of eosinophils suggesting that these cells are possibly not required for killing of the larvae in the adaptive immune response. This observation was also substantiated by earlier reports wherein it has been stated that a protective immune response utilizes different granulocytes in innate and adaptive protective immunity and eosinophils are required in the innate response i.e during a primary infection but not in adaptive response i.e secondary infection [57–59].

It was also noticed that pcD-Myo preferentially induced rapid expansion of CD8+T cells while Bm-Myo and pcD-Myo+Bm-Myo prime boost considerably up-regulated both CD4+ and CD8+ T cells along with B cell population. The production of pro-inflammatory (IL-2, IFN-γ) and anti-inflammatory (IL-4, IL-10) cytokines was highest in pcD-Myo+Bm-Myo vaccination showing a robust mixed Th1/Th2 type of immune response. Studies undertaken earlier by other workers [60–61] have demonstrated natural killer cell (NKT) activation by intradermal DNA priming as well as dendritic cell maturation leading to enhanced CD4 and CD8 T cell effector response and both these responses have been demonstrated to be important for the control of filariaisis. The above reports substantiate our findings where pcD-Myo primed antigen specific T-cells and then induced a rapid T-cell expansion with repeated antigen leading to finer protection by pcD-Myo+Bm-Myo vaccination group which illustrated more robust immune response than the pcD-Myo group.

Thus, it may be concluded that the current efforts to improve the efficacy of the Bm-Myo using alternative prime boost approach was successful. The data also revealed that the response elicited by homologous DNA vaccine was not as efficient as the recombinant myosin protein, however, DNA prime+protein-boost vaccination generated both humoral and cellular immune responses against *B*. *malayi* L3 unlike other immunization protocols which was capable of providing ~79% protection to BALB/c mice against challenged L3. The heterologous prime boost method of vaccination is therefore being advocated as a promising means of immunization against LF.

## Supporting Information

S1 Fig Schematic representation of (a) homologous DNA vaccination (pcD-Myo); (b) protein vaccination (Bm-Myo) and (c) heterologous prime boost regimen (pcD-Myo+Bm-Myo) in BALB/c mice. Animals in the DNA vaccination group received 100 μg pcD-Myo construct alone intradermally; in protein vaccination regimen mice received 25μg Bm-Myo with FCA subcutaneously and boosted with Bm-Myo emulsified in FIA, in heterologous prime boost regimen mice were primed with 100 μg pcD-Myo construct intradermally and two times boosted with 25μg Bm-Myo+FIA. ^a^Immunized Intradermally; ^b^Immunized subcutaneously.
(TIF)

S2 Fig Schematic representation of control mice immunized with different preparations. i) control group for pcD-Myo group; ii) Adjuvant group as control against Bm-Myo group; iii) mice immunized with vector pcDNA+Adjuvant as control group against pcD-Myo+BmMyo ^a^ Immunized Intradermally; ^b^Immunized subcutaneously.
(TIF)

S1 Table Percentage of different peritoneal leukocyte population after immunization and post L3 challenge in all animal groups. Percentages (mean±SE values) of different leukocytes were estimated by flow cytometry after immunization and post L3 challenge in all immunized. Statistical difference between mean values of each cell population post-immunization and post-L3 challenge in each individual group are shown as * *P*<0.05 and *** *P*< 0.001. Populations of all cells were increased significantly after L3 challenge in pcD-Myo+Bm-Myo group of mice.
(DOCX)
