# Supplementary figures and images for: Immunogenicity and Protective Efficacy of Brugia malayi Heavy Chain Myosin as Homologous DNA, Protein and Heterologous DNA/Protein Prime Boost Vaccine in Rodent Model
Source: PLoS One. 2015 Nov 11;10(11):e0142548. doi: 10.1371/journal.pone.0142548 (PMC4641661; doi:10.1371/journal.pone.0142548)

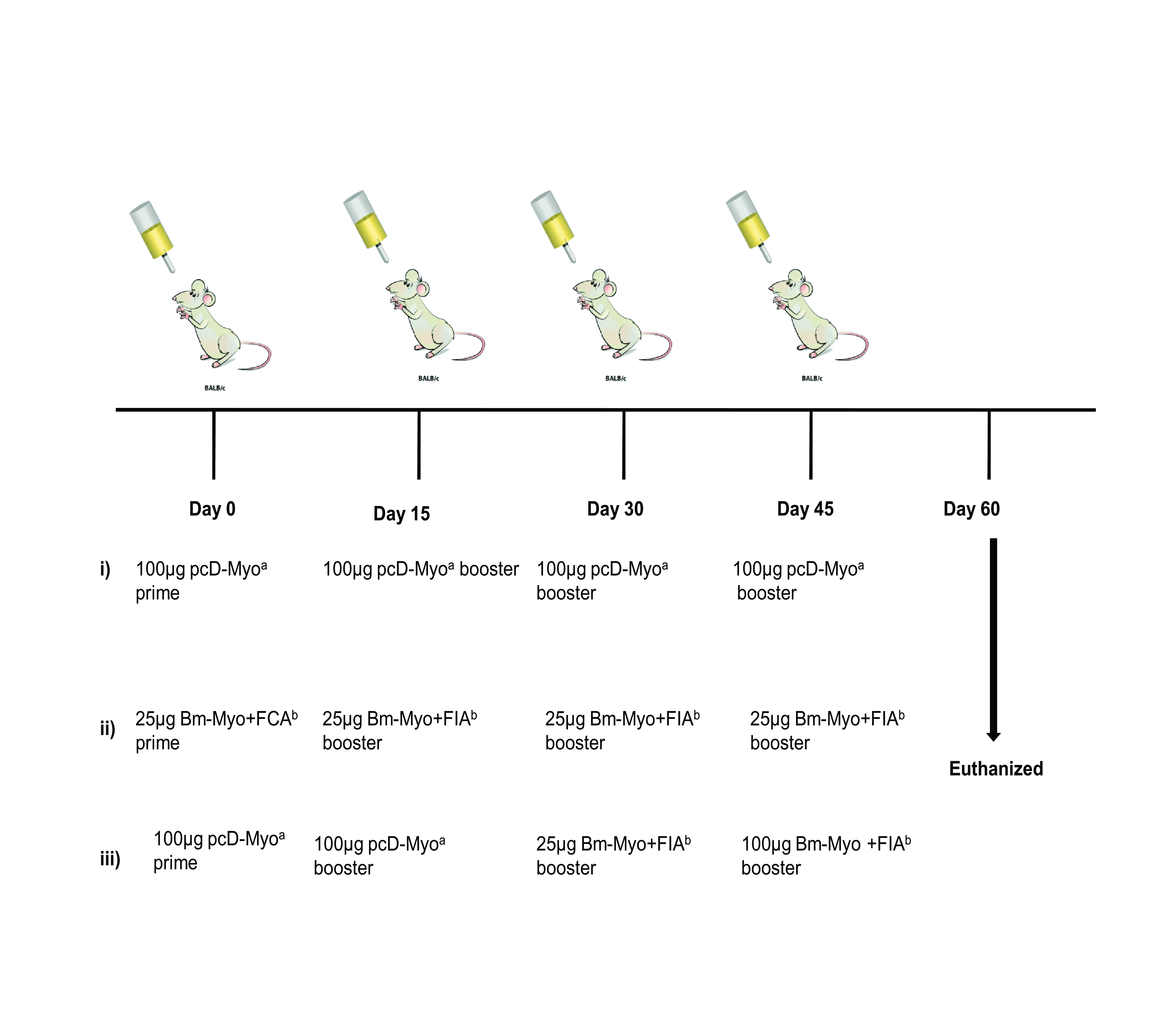

Supplement: S1 Fig — Animals in the DNA vaccination group received 100 μg pcD-Myo construct alone intradermally; in protein vaccination regimen mice received 25μg Bm-Myo with FCA subcutaneously and boosted with Bm-Myo emulsified in FIA, in heterologous prime boost regimen mice were primed with 100 μg pcD-Myo construct intradermally and two times boosted with 25μg Bm-Myo+FIA. aImmunized Intradermally; bImmunized subcutaneously. (TIF) [file pone.0142548.s001.tif]

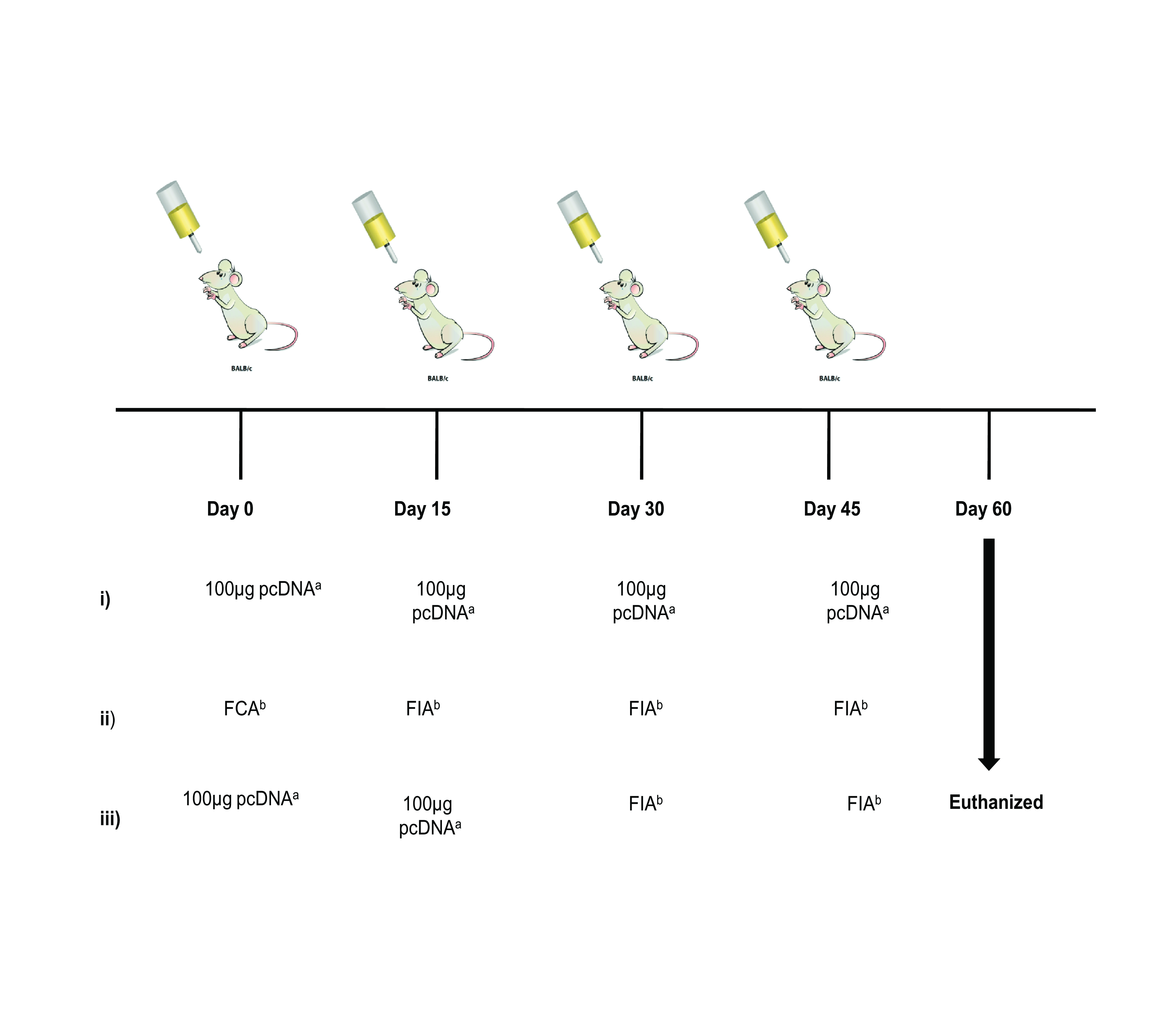

Supplement: S2 Fig — i) control group for pcD-Myo group; ii) Adjuvant group as control against Bm-Myo group; iii) mice immunized with vector pcDNA+Adjuvant as control group against pcD-Myo+BmMyo a Immunized Intradermally; bImmunized subcutaneously. (TIF) [file pone.0142548.s002.tif]
