# Supplementary material for: Immunogenicity and Protective Efficacy of Brugia malayi Heavy Chain Myosin as Homologous DNA, Protein and Heterologous DNA/Protein Prime Boost Vaccine in Rodent Model
Source: PLoS One. 2015 Nov 11;10(11):e0142548. doi: 10.1371/journal.pone.0142548 (PMC4641661; doi:10.1371/journal.pone.0142548)
Supplement: S1 Table — Percentages (mean±SE values) of different leukocytes were estimated by flow cytometry after immunization and post L3 challenge in all immunized. Statistical difference between mean values of each cell population post-immunization and post-L3 challenge in each individual group are shown as * P<0.05 and *** P< 0.001. Populations of all cells were increased significantly after L3 challenge in pcD-Myo+Bm-Myo group of mice. (DOCX) [file pone.0142548.s003.docx]

| **Leukocytes** | **Time point** | **% cell population (mean±SE)** | | | |
| --- | --- | --- | --- | --- | --- |
|  |  | **Control** | **Myo-pcD** | **Bm-Myo** | **Myo-pcD+Bm-Myo** |
| **Neutrophils** | Immunization | 8.2±0.73 | 6.7±0.60 | 9.8 ± 0.44 | 10.67±1.45 |
|  | L3 challenge | 7.34±0.60 | 7.17±1.04 | 11.47 ±0.51 | 16.37±0.73** |
| **Eosinophils** | Immunization | 31.33±1.76 | 29.6±0.88 | 29.00± 1.52 | 30.37±1.70 |
|  | L3 challenge | 46.67±2.4*** | 32±0.58 | 33 ±0.58 | 35.7±1.1* |
| **Macrophages** | Immunization | 61.67±1.16 | 61.78±1.21 | 66.57±1.15 | 69.34±0.88 |
|  | L3 challenge | 66.67±3.53 | 66.33±1.20 | 70.33±0.88 | 75.40±1.78* |
| **B cells** | Immunization | 10.82±1.00 | 13.77±0.87 | 18.33±1.20 | 22.93±0.64 |
|  | L3 challenge | \|  \|  \|  \|  \| \| --- \| --- \| --- \| --- \|   10.83±0.46 | 14.08±0.65 | 19.20±0.75 | 27.43±1.01* |
| **T cells** | Immunization | 19.30±0.67 | 20.67±0.74 | 25.00±0.71 | 29.57±0.83 |
|  | L3 challenge | 21.10±0.78 | 23.00±1.12 | 28.43±0.63 | 34.90±0.87** |

**P*<0.05 (low significance); ***P*<0.01( high significance); ****P*<0.001(very high significance)
